# Substitution Models of Water for Other Beverages, and the Incidence of Obesity and Weight Gain in the SUN Cohort

**DOI:** 10.3390/nu8110688

**Published:** 2016-10-31

**Authors:** Ujué Fresán, Alfredo Gea, Maira Bes-Rastrollo, Miguel Ruiz-Canela, Miguel A. Martínez-Gonzalez

**Affiliations:** 1 Department of Preventive Medicine and Public Health, University of Navarra, Medical School, Irunlarrea 1, 31008 Pamplona, Spain; ufresan@unav.es (U.F.); ageas@unav.es (A.G.); mbes@unav.es (M.B.-R.); mcanela@unav.es (M.R.-C.); 2 Navarra Institute for Health Research (IdisNa), 31008 Pamplona, Spain; 3 CIBER Physiopathology of Obesity and Nutrition (CIBERobn), Carlos III Institute of Health, 28029 Madrid, Spain

**Keywords:** Mediterranean cohort, water, soft drinks, beer, obesity, body weight

## Abstract

Obesity is a major epidemic for developed countries in the 21st century. The main cause of obesity is energy imbalance, of which contributing factors include a sedentary lifestyle, epigenetic factors and excessive caloric intake through food and beverages. A high consumption of caloric beverages, such as alcoholic or sweetened drinks, may particularly contribute to weight gain, and lower satiety has been associated with the intake of liquid instead of solid calories. Our objective was to evaluate the association between the substitution of a serving per day of water for another beverage (or group of them) and the incidence of obesity and weight change in a Mediterranean cohort, using mathematical models. We followed 15,765 adults without obesity at baseline. The intake of 17 beverage items was assessed at baseline through a validated food-frequency questionnaire. The outcomes were average change in body weight in a four-year period and new-onset obesity and their association with the substitution of one serving per day of water for one of the other beverages. During the follow-up, 873 incident cases of obesity were identified. In substitution models, the consumption of water instead of beer or sugar-sweetened soda beverages was associated with a lower obesity incidence (the Odds Ratio (OR) 0.80 (95% confidence interval (CI) 0.68 to 0.94) and OR 0.85 (95% CI 0.75 to 0.97); respectively) and, in the case of beer, it was also associated with a higher average weight loss (weight change difference = −328 g; (95% CI −566 to −89)). Thus, this study found that replacing one sugar-sweetened soda beverage or beer with one serving of water per day at baseline was related to a lower incidence of obesity and to a higher weight loss over a four-year period time in the case of beer, based on mathematical models.

## 1. Introduction

Obesity is a major epidemic in the 21st century for developed countries. In fact, 20%–30% of the Western adult population is obese [1], and the United States or some European countries have unacceptably high mean values of body mass index (BMI) [2]. In the last decade, its prevalence has risen seriously [3], and, although it is predicted to plateau by 2033, if the actual trend continues, around 30% of USA population would be overweight and obese [4]. These huge figures require new preventive measures and policy actions [4,5], as obesity is a risk factor for many chronic diseases such as cardiovascular disease, diabetes, some types of cancer and all-cause mortality [6]. Obesity is a multifactorial disorder [7,8]. Although sedentary lifestyle and epigenetics contribute to obesity, excessive caloric intake is a key determinant that needs to be addressed [9]. 

Beverages are major components of the daily diet. As for food, there are guidelines for beverage consumption in order to contribute to healthy diet [10,11]. Beverages can account for a substantial share of daily calories, even having low nutritional value, as it is the case of regular soft drinks and alcoholic beverages [12,13]. Solid and liquid preloads have been described as incomplete energy compensations [14], but beverages have a weaker satiety capacity than solids. Thus, a subsequent decompensated adjustment of calories intake takes place, causing an increase in total energy intake. Some beverages, like sugar-sweetened soda, are associated with weight gain and obesity [15,16]. Assessing alcoholic drinks, the relationship with these outcomes seems to depend on the type of alcohol analyzed because wine, beer and spirits may have different effects [17]. Water consumption has various health benefits, and a promising target for health promotion for obesity prevention could be to increase water intake at the expense of decreasing the consumption of other beverages [18,19].

Our objective was to evaluate the effect of substituting a serving per day of water for one of another beverage, or group of beverages according to the Spanish Society of Community Nutrition (Sociedad Española de Nutrición Comunitaria; SENC) recommendations, on obesity incidence and weight change in a Mediterranean cohort, using mathematical models.

## 2. Materials and Methods

### 2.1. Study Population

The Spanish project Seguimiento Universidad de Navarra (University of Navarra Follow-Up) (SUN) is a multipurpose, dynamic and prospective cohort, designed to establish relationships between diet and chronic conditions, such as obesity. All the participants are university graduates. Recruitment started in December 1999, and is permanently open. When participants are invited to enter the study, they receive, with the baseline questionnaire, a letter explaining the methodology, aims, data management and all information about the SUN cohort, including how to withdraw from the study. Informed consent was implied by the voluntary completion of the baseline questionnaire. Every two years, information from participants is collected by mailed or e-mailed questionnaires. When participants do not return a questionnaire, we send them a short exit questionnaire. The Research Ethics Committee of the University of Navarra approved the study. Further details of the study design and methods have been published elsewhere [20].

Up to March 2013, 21,686 participants were recruited. Among them, we excluded 2046 participants with total energy intake beyond predefined limits (<800 Kcal/day and <500 Kcal/day or >4000 Kcal/day and >3500 Kcal/day in men and women, respectively [21])—260 women who were pregnant at baseline or declared it in the second questionnaire, 1096 participants with a prevalent chronic disease such as cancer, diabetes and cardiovascular disease, and 513 participants with missing values in variables of interest in the analyses. Furthermore, 1706 people failed to answer the follow-up questionnaires (retention in the cohort: 90.7%), leaving a total of 16,065 participants. Finally, as this study was investigating the effect of beverage substitution on the incidence of obesity over time, we furthermore excluded people with prevalent obesity at baseline (*n* = 300). Therefore, the final number of participants for this analysis was 15,765.

### 2.2. Beverage Exposure Assessment

A semi-quantitative food frequency questionnaire (FFQ) was included in the baseline questionnaire. It was previously validated in Spain and recently re-evaluated [22,23]. The FFQ contained 17 beverage items (whole milk, reduced-fat milk, skim milk, milk shake, red wine, other kind of wine, beer, spirits, sugar-sweetened soda beverages (SSSBs), diet soda beverages, regular coffee, decaffeinated coffee, fresh orange juice, fresh non-orange fruit juice, bottled juice (any kind of fruit), tap water and bottled water). For each of them, frequencies of consumption were measured in nine categories, ranking from never/almost never to >6 servings/day. Serving size differed between beverages: coffee = 50 mL, wine = 100 mL, beer = 330 mL, spirits = 50 mL and, for the remaining beverages, a serving was equivalent to 200 mL.

All beverages reported were grouped according to SENC recommendations [11] and other publications [24] into six groups: two items on water (tap and bottled water), three items on low/non-caloric beverages (LNCBs) (non-sugared coffee (decaffeinated and regular) and diet soda beverages), nine items on milk, juice and sugared coffee (whole, reduced-fat and skim milk, milk shake, fresh orange and non-orange fruit juice, and any kind of fruit bottled juice, and sugared coffee (decaffeinated and regular), two items on occasional consumption (SSSBs and spirits), two items on wine (red and other kind of wine) and one item on beer (beer). The SENC has put together beverages into groups according to the evidence of quantity of energy and nutrients, benefits and harmful effects, and hydration capacity of each beverage. Liquids consumed as part of a food item are not taken into account. Our questionnaire did not distinguish between coffee with or without sugar. To make this distinction, we assumed that if the sugar intake was equal to or bigger than servings of coffee (both the decaffeinated and the regular one), coffee was drunk with sugar. Conversely, if sugar consumption was smaller than servings of coffee, coffee was assumed to be taken without sugar.

### 2.3. Outcome Assessment

Weight information was self-reported at baseline and in the follow-up questionnaires every two years. BMI was calculated as weight in kilograms divided by the square of height in meters. The validity of these measures has been assessed in a subsample of this cohort [25]. The mean relative error in self-reported weight was 1.45%, and the correlation coefficient between measured and self-reported weight was 0.99 (95% confidence intervals (95% CI) 0.98 to 0.99). For BMI, the mean relative error was 2.64% with a correlation coefficient of 0.94 (95% CI 0.91 to 0.97) [25]. The outcomes were incidence of obesity and weight change. A participant was classified as an incident case of obesity if his/her BMI was lower than 30 kg/m^2^ at baseline and equal to or higher than 30 during the follow-up. Average change in body weight was assessed between baseline and the four-year follow-up questionnaire, subtracting the first from the second.

### 2.4. Assessment of Other Variables

The baseline questionnaire also inquired about socio-demographic factors, medical history, and health-related habits. To quantify physical activity during free time, we assessed time spent in 17 activities at baseline, in order to compute an activity metabolic equivalent index (MET). Each activity was assigned a multiple of resting metabolic rate (MET score) [26] and time spent in each activity was multiplied by its specific MET score. Self-reported weekly MET-h correlated with energy expenditure objectively measured in a subsample of the cohort (Spearman *r* = 0.51; 95% CI 0.232 to 0.707) [27]. Adherence to Mediterranean diet was evaluated using the nine-item Mediterranean diet score developed by Trichopoulou and colleagues [28]. When the beverage that we were analyzing was included in this score, we recalculated it after excluding the item that we were studying, to avoid overlapping with the main exposure.

### 2.5. Statistical Analyses

We evaluated the association between substituting one serving per day of water for each beverage or beverage group (increasing one serving of water and decreasing one serving of the beverage/group in question) and incident obesity using mathematical models [29]. These replacements referred only to reported consumption at baseline; changes in beverage intake over time were not assessed. We fitted generalized estimating equations (GEE) models to evaluate the association of the described substitutions with obesity incidence. We assumed a binomial distribution, a logit link function, and an exchangeable correlation matrix. All completed observations from each participant were included, from the baseline to either the questionnaire in which the participant was classified as an incident case of obesity or the last follow-up questionnaire. Data received from participants after their classification as an incident case of obesity were excluded. As mentioned before, exposure was assumed constant for this model. If women reported a pregnancy during follow-up were censored at the questionnaire previous to their pregnancy. The Odds Ratio (OR) and 95% CI were estimated as the difference between β coefficients of exchanged beverages and then exponentiated [29]. Linear regression models were used to assess the association between the beverage replacements and four-year weight change. We estimated the adjusted absolute mean weight change (and 95% CI) of the beverage substitutions as the difference between β of exchanged beverages [29]. We fitted a crude univariate model, an age- and sex-adjusted model, and a multiple-adjusted model adjusted for the following potential confounders: sex, age, age squared, baseline BMI (kg/m^2^), physical activity (MET-h/week), smoking habit (never smoker, current smoker, former smoker), personal and family history of obesity, following a special diet, adherence to the Mediterranean dietary pattern, snacking between meals, weight change during the five years prior to baseline, and total energy intake from other sources than the exchanged beverages. When the analyses were carried out for group of beverages, we additionally adjusted for servings per day of other groups. Interactions were assessed using the Wald test for the two product terms between each beverage involved in the substitution and the characteristic evaluated.

In order to calculate the contribution of each beverage (or group of them) to the between-person variability in fluid intake, we conducted nested regression analyses after a stepwise selection algorithm. The contribution of each beverage is shown in the cumulative *R*^2^ change. Furthermore, we estimated their contribution related to total fluid intake as the mL consumed from each beverage divided by total fluid intake (%).

To ensure that the method of dealing with missing values did not influence the results, we performed a sensitivity analysis using multiple imputation technique to impute missing values in weight during follow-up. We imputed weight change over four years according to sex, age, BMI, physical activity, smoking status, if a special diet was followed, adherence to Mediterranean diet and snacking between meals, generating 20 complete datasets. Furthermore, we refitted the models in different sensitivity analyses to assess the robustness of our results: excluding participants who answered less than 10% of beverage items; excluding participants with weight change in previous five years due to pregnancy; excluding participants with personal history of obesity; excluding participants with family history of obesity; excluding participants with baseline BMI ≥ 27.5 kg/m^2^; excluding participants with a total energy intake under or over limits of daily calorie requirements, which is the basal metabolic rate (BMR) value multiplied by a factor depending on the activity level. We excluded people under BMR*1.2 and/or over BMR*1.9. BMR was estimated with the Mifflin–St Jeor equation [30]. Analyses were repeated after stratifying by sex, age (under or over the median) or physical activity (under or over the median of MET-h/week). Finally, we refitted the analysis using Cox regression. Hazard ratios (HRs) and 95% CI were estimated as the difference between β coefficients of exchanged group of beverage and then exponentiated [29]. All *p*-values presented are two-tailed; *p* < 0.05 was considered statistically significant. Analyses were performed using STATA/SE V.12.1 (StataCorp, College Station, TX, USA).

## 3. Results

Our analysis included a total of 16,065 participants (6455 men and 9610 women). The principal baseline characteristics of participants across quintiles of water consumption are presented in Table 1. The median water intake was five servings per day, and the interquartile range was 2.5–7; these are equivalent to 1000 mL, 500–1400 mL, respectively. The mean age of the sample was 37.9 years (standard deviation (SD): 11.7) and the mean BMI was 23.49 kg/m^2^ (SD: 3.5). Participants in the fifth quintile of water consumption compared to those in the first quintile were more likely to be women, younger and with a personal and/or family history of obesity; more participants in the top quintile of water intake had lost weight in the previous five years and their total energy intake was higher; on average, they consumed snacks between main meals more frequently, they were more likely to have followed a special diet and had better adherence to Mediterranean diet; they had higher fibre intake and their intake of almost every nutrient analyzed was higher, except for alcohol, which was slightly smaller; they were more active, spent less time having a sleeping siesta and were less prone to be a former smokers than those in the first quintile. According to other beverage consumption, they drank more servings of beverages included in LNCBs, spirits, and milk, juice and sugared coffee groups, and less SSSBs and wine, although the differences were small.

There were 873 incident cases of obesity during the follow-up. The incidence of obesity was estimated according to the substitution of one of the beverages gathered in the questionnaire by one glass of water per day, in crude and multivariable-adjusted models (Table 2). The substitution of beer with water was associated with a lower incidence of obesity (OR 0.81 (95% CI 0.69 to 0.94)). The association was also significant in the case of SSSBs (OR 0.85 (95% CI 0.75 to 0.97)). 

**Figure 1:**
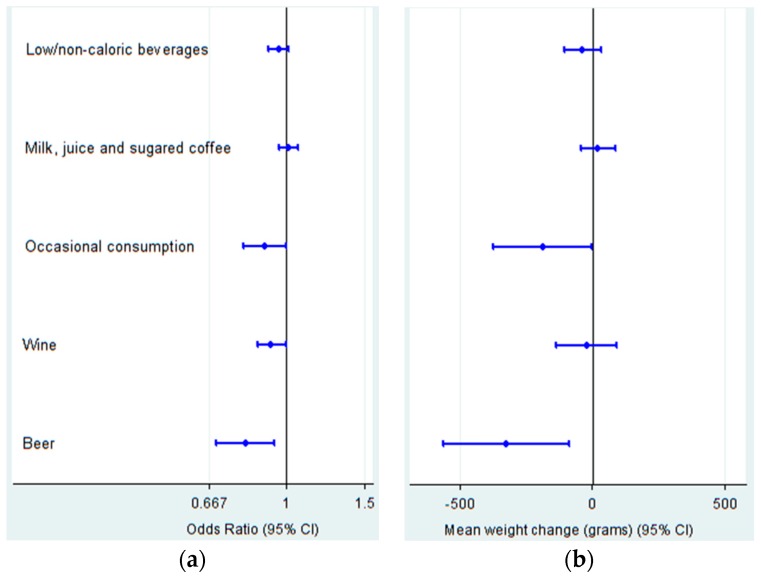
Substitution for group of beverages of one serving/day of water at baseline, using mathematical models. Low/non-caloric beverages contains: non-sugared coffee (decaffeinated and regular) and diet soda beverages; Milk, juice and sugared coffee contains whole, reduced-fat and skim milk, milk shake, fresh orange and non-orange fruit juice, and any kind of fruit bottled juice, and sugared coffee (decaffeinated and regular); Occasional consumption contains sugar-sweetened soda beverages and spirits; (**a**) the Odds Ratio (OR) (95% confidence interval (CI)) for incident obesity; and (**b**) four-year mean absolute weight change (g) (95% CI). Multiple-adjusted model.

Figure 1a shows the assessment of the incidence of obesity depending on the substitution of water for beverage groups made according to SENC recommendations. In the multiple-adjusted model, we observed an 11% lower incidence of obesity for the group of occasional consumption (SSSBs and spirits) (OR 0.89 (95% CI 0.80 to 0.99)), 8% lower for the group of wine (OR 0.92 (95% CI 0.86 to 0.99)) and 19% lower for beer (OR 0.81 (95% CI 0.69 to 0.94)).

When we estimated the odds ratio additionally adjusted for the consumption of other beverage groups, the statistical significance was maintained for the beer group (OR 0.84 (95% CI 0.71 to 0.98)) but it was no longer significant for the group of SSSBs and spirits (OR 0.92 (95% CI 0.82 to 1.03)) or wine (OR 0.94 (95% CI 0.87 to 1.02)). We did not observe any significant association with obesity for people in the fifth quintile of water consumption versus those in the first quintile (OR 1.03 (95% CI 0.82 to 1.30)).

SENC recommends the use of water or, if not, low/non-caloric beverages instead of caloric options. For that reason, we performed the same analysis but replacing by LNCBs. When low/non-caloric options were assumed to be used to replace a serving of beer, this change was associated with a lower incidence of obesity (OR 0.84 (95% CI 0.71 to 1.00) *p* = 0.05), but this was not observed for the substitution of LNCBs neither for SSSBs and spirits (OR 0.94 (95% CI 0.83 to 1.06)) nor for wine (OR 0.96 (95% CI 0.88 to 1.05)).

Table 3 shows the absolute four-year mean weight change (g) associated with substituting each beverage by a serving/day of water. The replacement of water for beer assumed a reduction of 328 g (95% CI −566 to −89). Refitting the models after using multiple imputations to impute missing values of weight change over four years, we obtained similar results, with the only statistically significant substitution being water for beer (−319 (−555 to −83)).

When performing the study for beverage groups, we observed the already described change in weight for reducing beer at the expense of increasing water. In the case of beverages recommended as occasional consumption (SSSBs and spirits), this substitution is also statistically significant, decreasing 187 g (95% CI −374 to 0; *p* = 0.05) (Figure 1b). When we additionally adjusted for the consumption of other beverage groups, the significance was maintained for beer (−308 (95% CI −550 to −65)), but not for SSSBs and spirits (−164 (95% CI −352 to 24)). We did not observe any relationship when analyzing the body weight change of participants in the fifth versus the lowest quintile of water consumption (−187 (95% CI −477 to 103)). Substitution of LNCBs for beer was associated with a reduction of 291 g in body weight in a four-year period (95% CI −535 to −47), but this association was not observed for the group of SSSBs and spirits (49 (95% CI −25 to 123)).

After analyzing the contribution of each beverage to the total intake of fluids, we concluded that water was the main source of fluid consumption among all beverage items (56.28%) and also the main source of variability (*R*^2^ = 0.715) in our population (Table 4).

We performed several sensitivity analyses in order to discard potential biases due to our assumptions and to test the robustness of our results (Table 5). When we refitted the analysis using Cox regression instead of GEE, we did not detect significant differences between the two models, but significance for wine group replacement was lost (HR: 0.96 (95% CI 0.89 to 1.04)). Results of the sensitivity analyses did not substantially change in any of these scenarios, except for the analysis of the incidence of obesity after stratifying the population according to sex and leisure-time physical activity (dichotomous: under or equal and over the median, 16.10 MET-h/week). We observed that the substitution of one serving of water for one of the group of SSSBs and spirits was significantly associated with a lower incidence of obesity in women (OR: 0.78 (95% CI 0.63 to 0.96)) but not in men (OR: 0.96 (95% CI 0.84 to 1.10)). However, the interaction was not statistically significant (*p* = 0.65). Furthermore, this replacement was associated with a lower incidence of obesity in those who were less active (OR: 0.83 (95% CI 0.73 to 0.95)) but not among participants who practiced more physical activity (OR: 1.02 (95% CI (0.82 to 1.26)). Again, the interaction was not statistically significant (*p* = 0.16). Stratifying by sex, there was a difference as well for the substitution for wine group (OR: 1.23 (95% CI 0.95 to 1.59) for women, and OR: 0.92 (95% CI 0.85 to 1.00) for men), but the interaction was not significant (*p* = 0.11). 

## 4. Discussion

The results from the present study indicate that replacing a serving of water for beer or sugar-sweetened soda beverages at baseline (using mathematical models) was associated with a lower incidence of obesity, and, in the case of beer, this potential intervention would reduce average weight in a four-year period. Furthermore, water was the main source of fluid consumption among all beverage items as well as the main source of variability in our population.

It is assumed that excessive alcohol consumption increases the risk of obesity because it is a source of energy per se and because energy from alcohol is not substitutive for the calories coming from food; instead, they are extra added calories [24]. Our results showed that the replacement of water for each group that contains alcoholic beverages (occasional consumption (SSSBs and spirits), wine and beer groups) was associated with a lower incidence of obesity. However, when we analyzed them separately, we only observed a statistically significant association for beer, but not for any kind of wine or spirits. Once again, the only one with a significant association with weight change was beer. In the literature, the evidence about the relationship between alcohol intake and body weight is contradictory [17]. In several investigations, a positive correlation has been described, while, in others, alcohol consumption was not related to body weight or to reducing the risk of weight gain and obesity. It seems that the positive association is more evident in heavy drinkers [31,32], whereas moderate consumption does not have an association [33], or it is negative [34]. Apart from the quantity of alcohol intake, it has been shown that not all types of alcoholic beverages have the same effect on body weight [35]. 

The positive correlation between beer consumption and weight gain and the risk of being overweight or obesity has been already published by our group for people whose BMI at baseline was lower than 25 kg/m^2^ [24]. However, in that study, the effect of beer was not analyzed alone, but with spirits. This time, we analyzed them separately due to their different effects on health. The SENC recommends alcohol-free beer intake due to its hydration capacity and because it is a source of vitamin B, fibre, minerals and antioxidants. Furthermore, it has been suggested that a moderate consumption of regular beer could be accepted in a healthy diet because moderate drinking may have some health benefits. For instance, a crossover study showed that moderate consumption of beer facilitates the recovery of the hormonal and immunological metabolism in active individuals after physical exercise [36].

Nothing is stated in the SENC recommendations about spirits. We decided to classify it in the occasional consumption group with SSSBs, allowing a maximum of one serving per week. We did not observe any correlation between the substitution for a serving of spirit with a serving of water neither in incidence of obesity nor in weight change. This finding may explain that the previously reported effect on weight gain and the risk of obesity by the group composed of beer and spirits could be attributable only to beer [24], and in the present study, to the effect of SSSBs, as was shown by analyzing sugared sodas separately. In fact, increasing beer consumption was associated with waist circumference in the prospective Copenhagen City Heart Study, and although they reported an association between moderate-to-large spirits consumption and high waist circumference in both sexes, they concluded that their result was non-conclusive because of the large CI [35]. In another Danish prospective cohort, consumption of spirits was statistically associated with higher waist circumference in women after five years of follow-up, but the absolute change did not have relevance from a practical point of view due to its small magnitude [37].

The SENC suggests that beer and wine consumption should both be moderate. However, deriving from our previous investigations where we did not observe a correlation between wine intake and changes on body weight or the risk of obesity [24], we decided to analyze it separately from beer. In fact, moderate consumption of wine, especially red wine, has been negatively or not associated with body weight. This lack of association (or its protector effect at lower levels of consumption [37]) has been explained not only because wine drinkers usually follow a healthier diet [38] (all of our analyses were adjusted for multiple covariates, including diet quality, decreasing the potential residual confounding) but also because of the inherent properties of wine due to its components [39,40]. Indeed, a clinical trial carried out in 14 healthy young men showed that the addition of two servings of wine per day over six weeks did not affect anthropometric parameters [41]. When we analyzed the replacement of water for red or other types of wine separately, one of them predicted the outcomes, although the substitution for red wine was close to the statistical significance for obesity incidence (OR 0.92 (95% CI 0.84 to 1.00); *p* = 0.062). Furthermore, for the replacement of a serving from the group of both types of wine, we found an association for obesity incidence, but not for weight change in a four-year period. In the Danish study previously described, they found a U-shape relationship between wine consumption and waist circumference [37], so maybe our results are due to the moderate-high consumption of wine in our sample. If the association between wine consumption and weight gain follows a U-shape, a substitution in lower levels would result in a different effect than a substitution for higher levels of consumption.

Many studies have examined the association between intake of SSSBs and later weight change, and most of them suggested that their consumption increases the risk of obesity [42]. We have previously demonstrated that, among people with a previous history of weight gain, those in the fifth quintile of SSSB consumption (more than three servings per week) increased by 60% the odds of weight gain during a 28.5 month follow-up when compared to those with the lowest quintile (never/almost never intake) [43]. Previous studies have analyzed the effect of the real substitution of water for a caloric soft drink, or the other way around. Short-term clinical trials have investigated the consequences of the replacement of SSSBs for water before or during meals [43]. They concluded that this change supposed an increase of 7.8% in total energy intake. With data from a 12-month “A TO Z” intervention, consisting of increasing water intake in substitution for SSSBs, this replacement was associated with lower energy intake in premenopausal overweight women [44], and a significant reduction of weight and fat [45]. However, it should be noted that this study was designed as a weight loss intervention; therefore, it was expected that energy intake and body weight would decrease. Our analyses did not show a correlation between water substitution for SSSBs and weight change in a four-year period, independently of total energy intake, but in the case of the group in which SSSBs were gathered, the association was just statistically significant (−187 g (95% CI −374 to 0; *p* for trend = 0.05)). Our results suggested that this replacement could be associated with a lower incidence of obesity. Olsen and Heitmann [42] informed readers that the most consistent studies that analyzed the association between calorically sugared beverages and body weight/obesity are those whose follow-up period is five years or more. This may be a potential reason why we could not appreciate in full the effect of the replacement for SSSBs on average body weight given that the follow-up analyzed was shorter than that recommended by these authors. In fact, when we studied the incidence of obesity over a longer period, the replacement of water for SSSB was correlated with the reduction in that risk. It was proposed that the mechanism by which the increase of this type of beverages in decrement of water affects body weight is the subsequent increase of energy intake. However, some studies that affirm a positive correlation between SSSBs and obesity do not present any differences in their results when data are adjusted for energy intake [42]. Thus, there must be more biological mechanisms that relate them, along with the increment of energy intake. Other studies proposed that the intake of SSSBs may fail to trigger physiological satiety mechanisms or that the consumption of this type of beverages may cause a lower thermogenesis, resulting in an increase of energy intake [42]. Furthermore, although the available data about the relationship between increasing water consumption and body weight is not very conclusive, it has been suggested that drinking water could control body weight by inducing thermogenesis [46]. This effect of water, combined with the decrease of SSSB intake, could give an explanation of the effect on the body via a thermogenesis pathway. We did not find any correlation between increasing water consumption at baseline either with less weight gain nor incidence of obesity. In fact, all of the studies which correlate water and body weight, although interesting, are based on short-term studies, thus they may not be applicable to long-term effects [43,45]. 

The SENC suggests always drinking water, or, if not, low/non-caloric beverages to control body weight. When we analyzed the effect of substituting one serving of low/non-caloric beverages for one serving of beer, we could observe a correlation with a decrease in body weight and in obesity incidence. However, in replacement of one serving of LNCBs for one of the group in which SSSBs are gathered, we have not seen any association nor with reduction of weight neither in the risk of obesity. It was also not observed for obesity incidence when the replacement was made for a wine group. Available evidence is not very clear about the topic of diet soda beverages and weight loss [19,47]. Studies about the effects of diet drink consumption could be affected by an unmeasured confounding factor (for example, people who prefer dieting instead of regular soft drinks may be healthier or perform other strategies that could influence in body weight). It is possible that the correlation between diet soda beverage intake and weight loss are only evident in overweight and obese people, as was reported in three American prospective cohorts [19], or even in clinical trials [48]. In randomized controlled trials in adults whose BMI exceeded the healthy value, the replacement of water or diet soft drinks for SSSBs achieved a 5% weight loss compared to the control group [49] and a reduction on total energy intake [50], as expected as part of a weight loss program. A longer follow-up trial with overweight and obese adolescents showed a reduction in BMI from replacing sugar-sweetened soda beverages with the diet version after one year, but not at the two-year follow-up [48]. This effect was also reported for healthy weight children in a double-blind, 18-month clinical trial, which showed that the intake of non-caloric soft drinks instead of the regular version was associated with lower weight gain, confirming that the replacements may have effects on body weight not only in overweight people but also in those with healthy weight at baseline [51]. On the other hand, water was not superior to non-nutritive sweetened beverages in a weight loss intervention trial [52]. Our cohort included people independently of their baseline BMI and the analysis was adjusted for total energy intake. More studies are needed before recommendations can be made to the general population regarding the consumption of diet soda drinks as a substitute of regular soft drinks instead of water.

We did not find any correlation between the replacement of water for any type of juice analyzed with weight change in a four-year period, nor with the incident of obesity. In a previous study, we found a 16% (OR 1.16 (95% CI 0.99 to 1.36)) increase in body weight when we compared people in the fifth quintile of sugared fruit juice consumption (six or more servings per week) with those in the first quintile (less than one serving per week) [53]. Three American cohorts concluded that the consumption of fruit juice increased weight, whereas its substitution by water caused a weight reduction [19]. However, these investigations did not take into account different types of juices. Only a few studies have analyzed the effect of fruit juice consumption on weight in adults, thus more are needed, in particular distinguishing between different types of juice [15]. 

Our analysis did not find any relationship between the substitution of water for any dairy product and weight gain or obesity. These findings are consistent with the results obtained from other cohorts [19,54,55] and a meta-analysis of randomized clinical trials [56], in which no correlation was found between dairy products consumption and weight change or obesity incidence.

The strengths of our study include its prospective design, which avoids the possibility of reverse causation bias potentially present in other types of studies, the previous validation of the questionnaires used, the use of a wide range score for beverage consumption, and a relatively large sample size. Additionally, we were able to control for multiple possible confounding variables and conducted various sensitivity analyses. The generalizability of the findings may be considered to be weak because the SUN cohort participants are all university graduates, and therefore the sample is not representative of the Spanish population. However, this enhanced the internal validity of our study because of the homogeneity of the population and the high education level and socioeconomic status, which reduces potential confounding.

Some potential limitations should be noted, as we used self-reported information. Although there is a tendency for participants to overestimate their height and underestimate their weight, self-reported weight and height was found to be valid in our cohort [25]. Beverage consumption was self-reported, and so it is susceptible to information bias. However, this method is arguably the best way to ascertain food habits in large cohorts that are followed over long periods [21]. Another limitation that should be taken into account is that the FFQ does not distinguish between coffee with or without sugar. We resolved this by considering that if the servings of sugar consumed per day are equal to or bigger than those of coffee (both the regular and decaffeinated kind), then that participant takes sugar-added coffee, and if the sugar servings are less than the coffee ones, then sugar-free coffee is taken. The item “bottle juice” does not specify between 100% fruit juice or juice from concentrates, or with or without added-sugar. Apart from that, our analysis was done using mathematical substitution models, thus real replacements may not show the same results. However, this technique has been widely used in nutritional epidemiology [29,57,58]. Furthermore, we have just measured baseline consumption, and not variations over time, thus changes in body weight could be a consequence of these disparities.

## 5. Conclusions

This study found that replacing one sugar-sweetened soda beverage (but not other sugared drinks, like fruit juices) or beer with one serving of water per day at baseline was related to a lower incidence of obesity and to a higher weight loss over a four-year period time in the case of beer, based on mathematical models. Nevertheless, longitudinal investigations based on real interventions are needed to confirm these potential effects. As obesity carries a high risk for the development of other diseases like diabetes or cardiovascular disease, the possible effects of the substitution for these beverages with water is an important target to consider in future public health research.

## Figures and Tables

**Table 1:** Distribution of baseline characteristics of participants across quintiles of water consumption ^1^.

	Quintiles of Water Consumption					
	Q1	Q2	Q3	Q4	Q5	*p*-Value *
*N*	5227	1457	3250	4000	2131	
Water intake ^1^ (mL)	357 (0, 500)	529 (513, 700)	1000 (1000, 1000)	1400 (1013, 1400)	1500 (1413, 2800)	<0.001
Sex (men %)	44.4	44.0	39.0	37.2	34.6	<0.001
Age (years)	40.8 (12.0)	38.3 (11.4)	38.2 (11.8)	35.8 (11.1)	34.1 (10.3)	<0.001
Baseline body mass index (kg/m^2^)	23.7 (3.42)	23.4 (3.33)	23.4 (3.50)	23.4 (3.49)	23.3 (3.53)	0.052
Current smoker (%)	21.8	20.9	20.4	21.8	22.9	<0.001
Former smoker (%)	31.1	26.1	29.4	26.6	25.2	<0.001
Personal history of obesity (%)	7.21	6.18	7.51	7.23	8.40	0.149
Family history of obesity (%)	21.6	22.9	23.0	24.3	23.0	0.047
Weight loss in the previous 5 years (%)	20.4	20.5	22.6	26.2	28.3	<0.001
Weight gain in the previous 5 years (%)	52.2	54.4	50.2	48.9	48.4	<0.001
Physical activity (MET-h/week)	19.1 (21.2)	20.6 (20.9)	21.1 (19.9)	23.8 (24.1)	25.8 (27.0)	<0.001
Total energy intake (kcal/day)	2233 (620)	2397 (602)	2369 (600)	2394 (596)	2430 (620)	0.047
Snacking between meals (%)	31.6	34.0	33.1	32.7	35.5	0.023
Following special diet (%)	6.62	5.97	7.23	8.18	10.09	<0.001
Adherence to Mediterranean diet (0–9)	3.98 (1.74)	4.10 (1.79)	4.14 (1.77)	4.30 (1.76)	4.40 (1.78)	0.690
Fat intake (g/day)	90.9 (30.7)	98.8 (30.9)	97.6 (30.4)	98.0 (30.5)	99.5 (31.5)	0.397
Saturated fatty acids intake (g/day)	31.5 (12.3)	33.9 (12.0)	33.1 (11.7)	33.0 (12.1)	33.6 (12.4)	0.025
Monounsaturated fatty acids intake (g/day)	38.7 (14.2)	42.3 (14.4)	42.0 (14.3)	42.2 (14.3)	42.8 (14.8)	0.383
Polyunsaturated fatty acids intake (g/day)	13.1 (5.72)	14.3 (5.94)	13.9 (5.91)	13.9 (5.80)	14.0 (5.69)	0.115
Carbohydrates intake (g/day)	244 (83.9)	262 (81.8)	259 (82.5)	263 (83.7)	265 (85.3)	0.362
Protein intake (g/day)	101 (28.0)	106 (26.4)	105 (26.5)	107 (27.3)	110 (28.9)	<0.001
Alcohol intake (g/day)	4.84 (9.03)	4.99 (7.59)	4.59 (7.80)	4.43 (7.62)	4.64 (6.94)	0.0734
Dietary fibre intake (g/day)	26.4 (12.2)	27.1 (11.2)	27.9 (11.8)	28.9 (12.5)	29.3 (12.4)	<0.001
Sleeping hours (h/day)	7.24 (0.91)	7.32 (0.78)	7.31 (0.80)	7.32 (0.84)	7.31 (0.82)	<0.001
Sleeping siesta (h/day)	0.34 (0.86)	0.29 (0.76)	0.28 (0.73)	0.30 (0.74)	0.27 (0.71)	<0.001
**Groups of beverages (servings/week)**						
**1. Water** ^#^	11.0 (6.69)	20.2 (2.72)	35.0 (0.00)	43.9 (6.03)	58.3 (13.86)	<0.001
**2. Low/non-caloric beverages**	7.01 (10.3)	7.49 (10.0)	7.21 (9.9)	7.39 (10.2)	7.77 (10.3)	0.105
Diet soda beverages ^#^	0.81 (3.22)	0.78 (2.79)	0.70 (2.58)	0.85 (2.70)	1.11 (3.54)	<0.001
Coffee without sugar ^†^	6.20 (9.52)	6.71 (9.47)	6.52 (9.34)	6.54 (9.63)	6.66 (9.50)	0.474
**3. Milk, juice and sugared coffee**	15.8 (11.4)	16.8 (10.9)	16.8 (10.6)	16.7 (11.4)	16.8 (11.7)	<0.001
Dairyproducts ^#^	9.33 (8.09)	9.79 (7.63)	9.58 (7.14)	9.72 (7.67)	9.71 (7.77)	<0.001
Juices ^#^	2.91 (4.29)	3.24 (4.13)	3.01 (3.86)	3.34 (4.83)	3.33 (4.60)	<0.001
Coffee with sugar ^†^	3.57 (6.37)	3.79 (6.44)	4.17 (6.66)	3.62 (6.42)	3.81 (7.06)	<0.001
**4. Occasional consumption**	2.04 (3.85)	2.09 (3.09)	1.83 (3.00)	1.83 (2.78)	2.02 (3.25)	<0.001
SSSBs ^#^	1.55 (3.41)	1.55 (2.65)	1.30 (2.29)	1.26 (2.11)	1.42 (2.87)	<0.001
Spirits ^†^	0.49 (1.35)	0.54 (1.21)	0.53 (1.34)	0.57 (1.46)	0.60 (1.17)	<0.001
**5.Wine** ^‡^	3.64 (6.45)	3.30 (5.75)	2.74 (5.31)	2.30 (5.01)	2.11 (4.55)	<0.001
**6. Beer** ^•^	1.34 (2.92)	1.37 (2.50)	1.25 (2.42)	1.14 (2.02)	1.23 (2.41)	<0.001

Mean and standard deviation (SD), or %. The SUN project 1999–2015. ^1^ Median and minimum and maximum; * Categorical variables were analyzed using X^2^ test and expressed as percentages. Continuous variables were analyzed using analysis of variance (ANOVA) test and expressed as means and SD otherwise indicated; ^#^ A serving of water, diet soda beverages, dairy products (whole, reduced-fat and skim milk, and milk shake), juices (fresh orange and non-orange fruit juice, and any kind of fruit bottled juice) and sugar-sweetened soda beverages (SSSBs) is defined as 200 mL; ^†^ A serving of any kind of coffee and spirits is defined as 50 mL; ^‡^ A serving of wine is defined as 100 mL. ^●^ A serving of beer is defined as 330 mL.

**Table 2:** The Odds Ratio (OR) (95% confidence interval (CI)) for incident obesity associated with the substitution of one serving/day of water for several beverages (increasing 1 serving/day of water and decreasing 1 serving/day of the beverage in question) at baseline, using mathematical models.

Substitution	Crude Model	Age- & Sex-Adjusted Model	Multiple-Adjusted Model ^1^
Water for beer	0.63 (0.55 to 0.71)	0.78 (0.67 to 0.91)	0.81 (0.69 to 0.94)
Water for SSSBs ^2^	0.80 (0.71 to 0.90)	0.82 (0.73 to 0.91)	0.85 (0.75 to 0.97)
Water for bottled juice	0.96 (0.78 to 1.19)	0.94 (0.79 to 1.13)	0.86 (0.73 to 1.02)
Water for diet soda beverages	0.77 (0.71 to 0.85)	0.75 (0.69 to 0.82)	0.91 (0.80 to 1.04)
Water for red wine	0.78 (0.72 to 0.84)	0.95 (0.87 to 1.04)	0.92 (0.84 to 1.00)
Water for other wines (non-red)	0.75 (0.64 to 0.87)	0.91 (0.76 to 1.10)	0.93 (0.76 to 1.13)
Water for skim milk	0.93 (0.86 to 1.00)	0.92 (0.86 to 0.99)	0.94 (0.87 to 1.03)
Water for whole milk	1.07 (0.97 to 1.18)	1.12 (1.00 to 1.24)	0.96 (0.87 to 1.06)
Water for regular coffee	0.89 (0.85 to 0.94)	0.94 (0.89 to 0.99)	0.97 (0.91 to 1.02)
Water for spirits	0.69 (0.55 to 0.85)	0.84 (0.67 to 1.04)	1.02 (0.77 to 1.34)
Water for decaffeinated coffee	0.87 (0.79 to 0.97)	0.93 (0.84 to 1.03)	1.05 (0.94 to 1.18)
Water for reduced-fat milk	1.10 (1.01 to 1.21)	1.08 (0.99 to 1.19)	1.06 (0.96 to 1.16)
Water for fresh non-orange fruit juice	1.09 (0.75 to 1.58)	1.13 (0.80 to 1.59)	1.06 (0.73 to 1.52)
Water for fresh orange juice	1.10 (0.93 to 1.31)	1.14 (0.97 to 1.33)	1.06 (0.90 to 1.24)
Water for milk shake	1.94 (0.89 to 4.25)	1.56 (0.83 to 2.97)	1.32 (0.79 to 2.22)

873 incident cases of obesity. ^1^ Additionally adjusted for baseline body mass index, physical activity, smoking habit, personal history of obesity, family history of obesity, following a special diet, adherence to the Mediterranean dietary pattern, snacking between meals, weight change in the past five years, and total energy intake from other sources than the exchanged beverages; ^2^ SSSBs: sugar-sweetened soda beverages.

**Table 3:** Mean four-year absolute weight change (95% CI) associated with the substitution of one serving/day of water for several beverages (increasing 1 serving/day of water and decreasing 1 serving/day of the beverage in question) at baseline, using mathematical models.

Substitution	Crude Model	Age- & Sex-Adjusted Model	Multiple-Adjusted Model ^1^
Water for milk shake	−554 (−1205 to 98)	−482 (−1134 to 171)	−399 (−1049 to 250)
Water for fresh non-orange fruit juice	−303 (−724 to 118)	−336 (−757 to 85)	−342 (−760 to 76)
Water for beer	−226 (−458 to 6)	−272 (−511 to −34)	−328 (−566 to −89)
Water for spirits	−265 (−695 to 166)	−274 (−713 to 165)	−226 (−667 to 216)
Water for SSSBs ^2^	−291 (−508 to −75)	−215 (−435 to 5)	−205 (−425 to 16)
Water for bottled juice	−203 (−469 to 63)	−172 (−437 to 94)	−137 (−400 to 127)
Water for diet soda beverages	−152 (−367 to 62)	−122 (−336 to 93)	−86 (−300 to 129)
Water for other wines (non-red)	86 (−270 to 441)	−24 (−382 to 335)	−41 (−397 to 315)
Water for red wine	60 (−75 to 195)	−24 (−167 to 119)	−38 (−181 to 104)
Water for regular coffee	−49 (−126 to 28)	−56 (−135 to 22)	−21 (−101 to 58)
Water for decaffeinated coffee	48 (−104 to 199)	−14 (−168 to 139)	5 (−148 to 157)
Water for reduced-fat milk	31 (−76 to 138)	31 (−76 to 138)	6 (−100 to 113)
Water for fresh orange juice	81 (−115 to 276)	43 (−153 to 239)	7 (−189 to 202)
Water for skim milk	52 (−57 to 160)	23 (−86 to 133)	28 (−82 to 137)
Water for whole milk	4 (−107 to 115)	20 (−92 to 132)	61 (−55 to 177)

^1^ Additionally adjusted for baseline body mass index, physical activity, smoking habit, personal history of obesity, family history of obesity, following a special diet, adherence to the Mediterranean dietary pattern, snacking between meals, weight change in the past five years, and total energy intake from other sources than the exchanged beverages; ^2^ SSSBs: sugar-sweetened soda beverages.

**Table 4:** Sources of variability (cumulative *R*^2^) and main sources (%) in total liquid intake.

Beverage	Cumulative *R*^2^	% of Total Liquid Intake
Water	0.715	56.28
Reduced-fat milk	0.740	6.90
Whole milk	0.765	6.78
Regular coffee	0.786	4.56
Skim milk	0.847	5.77
Bottled juice	0.861	1.71
Fresh orange juice	0.891	3.93
Diet soda beverage	0.914	1.57
SSSBs ^1^	0.933	3.07
Beer	0.978	4.26
Decaffeinated coffee	0.981	0.96
Red wine	0.992	2.10
Milk shake	0.994	0.54
Fresh non-orange fruit juice	0.998	0.84
Another type of wine (non-red)	0.999	0.43
Spirits	1.000	0.30

^1^ SSSBs: sugar-sweetened soda beverages.

**Table 5:** Sensitivity analyses. OR (95% CI) for incident obesity associated with the substitution of beverages by one serving/day of water.

	Cases	Low/Non-Caloric Beverages ^1^	Milk, Juice and Sugared Coffee ^2^	Occasional Consumption ^3^	Wine	Beer
Overall	873	0.96 (0.91 to 1.01)	1.01 (0.96 to 1.06)	0.89 (0.80 to 0.99)	0.92 (0.86 to 0.99)	0.81 (0.69 to 0.94)
Excluding participants who answered ≤10% beverage items	862	0.95 (0.91 to 1.00)	1.00 (0.95 to 1.05)	0.89 (0.80 to 1.00)	0.92 (0.85 to 0.99)	0.80 (0.69 to 0.94)
Excluding participants with weight change in the previous 5 years due to pregnancy	854	0.96 (0.91 to 1.02)	1.01 (0.96 to 1.06)	0.90 (0.80 to 1.01)	0.92 (0.86 to 1.00)	0.80 (0.68 to 0.95)
Excluding participants with personal history of obesity	623	0.93 (0.87 to 0.98)	1.03 (0.97 to 1.09)	0.91 (0.81 to 1.02)	0.92 (0.84 to 1.01)	0.76 (0.64 to 0.89)
Excluding participants with family history of obesity	587	0.96 (0.90 to 1.01)	1.03 (0.97 to 1.10)	0.96 (0.84 to 1.09)	0.93 (0.85 to 1.01)	0.81 (0.67 to 0.97)
Excluding participants with BMI ≥ 27.5	369	0.94 (0.87 to 1.01)	1.04 (0.96 to 1.12)	0.89 (0.77 to 1.03)	0.90 (0.80 to 1.02)	0.77 (0.61 to 0.96)
Energy limits: under or over limits of daily calories needs, according to BMR ^‡^	441	0.98 (0.91 to 1.05)	0.92 (0.86 to 0.98)	0.79 (0.69 to 0.90)	0.87 (0.78 to 0.97)	0.81 (0.67 to 0.98)
Assessing only women	358	0.97 (0.89 to 1.05)	1.01 (0.93 to 1.09)	0.78 (0.63 to 0.96) ^†^	1.23 (0.95 to 1.59) ^¥^	0.71 (0.42 to 1.20)
Assessing only men	515	0.96 (0.90 to 1.03)	1.00 (0.94 to 1.07)	0.96 (0.84 to 1.10) ^†^	0.92 (0.85 to 1.00) ^¥^	0.79 (0.68 to 0.91)
Assessing only people under 35 years old	281	0.92 (0.85 to 1.00)	1.06 (0.98 to 1.16)	0.90 (0.75 to 1.08)	0.83 (0.67 to 1.01)	0.72 (0.56 to 0.94)
Assessing only people 35 year olds or older	592	0.98 (0.92 to 1.05)	0.99 (0.93 to 1.05)	0.90 (0.78 to 1.03)	0.92 (0.85 to 1.01)	0.84 (0.70 to 1.02)
Assessing only less active people (under the median)	500	0.95 (0.89 to 1.01)	1.03 (0.97 to 1.10)	0.83 (0.73 to 0.95) *	0.93 (0.84 to 1.04)	0.82 (0.67 to 1.02)
Assessing only more active people (in and over the median)	373	0.97 (0.90 to 1.06)	0.98 (0.91 to 1.05)	1.02 (0.82 to 1.26) *	0.89 (0.80 to 0.99)	0.79 (0.63 to 0.99)

Adjusted for sex, age, age squared, baseline body mass index (BMI), physical activity, smoking habit, personal history of obesity, following a special diet, adherence to the Mediterranean dietary pattern, snacking between meals, weight change in the past 5 years, and total energy intake from other sources than the exchanged beverages. ^1^ Non-sugared decaffeinated/regular coffee and diet soda beverages; ^2^ Any king of juice and dairy product, and sugared decaffeinated/regular coffee; ^3^ Sugar-sweetened soda beverages and spirits; ^†^ *p* for interaction = 0.6527; ^¥^ *p* for interaction = 0.1145; * *p* for interaction = 0.198; ^‡^ The daily calorie needs is the basal metabolic rate value multiplied by a factor with a value between 1.2 and 1.9, depending on the activity level. The basal metabolic rate (BMR) is estimated with the Mifflin–St Jeor equation.

## References

[1] Flegal K.M., Carroll M.D., Ogden C.L., Johnson C.L. (2002). Prevalence and trends in obesity among us adults, 1999–2000. JAMA.

[2] World Health Organization (2007). The Challenge of Obesity in the WHO European Region and the Strategies for Response.

[3] Stevens G.A., Singh G.M., Lu Y., Danaei G., Lin J.K., Finucane M.M., Bahalim A.N., McIntire R.K., Gutierrez H.R., Cowan M. (2012). National, regional, and global trends in adult overweight and obesity prevalences. Popul. Health Metr..

[4] Thomas D.M., Weedermann M., Fuemmeler B.F., Martin C.K., Dhurandhar N.V., Bredlau C., Heymsfield S.B., Ravussin E., Bouchard C. (2014). Dynamic model predicting overweight, obesity, and extreme obesity prevalence trends. Obesity.

[5] Swinburn B.A., Sacks G., Hall K.D., McPherson K., Finegood D.T., Moodie M.L., Gortmaker S.L. (2011). The global obesity pandemic: Shaped by global drivers and local environments. Lancet.

[6] Guh D.P., Zhang W., Bansback N., Amarsi Z., Birmingham C.L., Anis A.H. (2009). The incidence of co-morbidities related to obesity and overweight: A systematic review and meta-analysis. BMC Public Health.

[7] Marti A., Moreno-Aliaga M.J., Hebebrand J., Martinez J.A. (2004). Genes, lifestyles and obesity. Int. J. Obes. Relat. Metab. Disord. J. Int. Assoc. Study Obes..

[8] Gesta S., Bluher M., Yamamoto Y., Norris A.W., Berndt J., Kralisch S., Boucher J., Lewis C., Kahn C.R. (2006). Evidence for a role of developmental genes in the origin of obesity and body fat distribution. Proc. Natl. Acad. Sci. USA.

[9] Hawkes C., Smith T.G., Jewell J., Wardle J., Hammond R.A., Friel S., Thow A.M., Kain J. (2015). Smart food policies for obesity prevention. Lancet.

[10] Popkin B.M., Armstrong L.E., Bray G.M., Caballero B., Frei B., Willett W.C. (2006). A new proposed guidance system for beverage consumption in the united states. Am. J. Clin. Nutr..

[11] Sociedad Española de Nutrición Comunitaria (2009). Guía para una hidratación saludable. La Declaración de Zaragoza. SENC, 2008. Rev. Esp. Nutr. Comunitaria.

[12] Wolf A., Bray G.A., Popkin B.M. (2008). A short history of beverages and how our body treats them. Obes. Rev. Off. J. Int. Assoc. Study Obes..

[13] Garriguet D. Beverage Consumption of Canadian Adults. http://www.statcan.gc.ca/pub/82-003-x/2008004/article/6500240-eng.htm.

[14] Almiron-Roig E., Palla L., Guest K., Ricchiuti C., Vint N., Jebb S.A., Drewnowski A. (2013). Factors that determine energy compensation: A systematic review of preload studies. Nutr. Rev..

[15] Malik V.S., Schulze M.B., Hu F.B. (2006). Intake of sugar-sweetened beverages and weight gain: A systematic review. Am. J. Clin. Nutr..

[16] Vartanian L.R., Schwartz M.B., Brownell K.D. (2007). Effects of soft drink consumption on nutrition and health: A systematic review and meta-analysis. Am. J. Public Health.

[17] Sayon-Orea C., Martinez-Gonzalez M.A., Bes-Rastrollo M. (2011). Alcohol consumption and body weight: A systematic review. Nutr. Rev..

[18] Muckelbauer R., Sarganas G., Gruneis A., Muller-Nordhorn J. (2013). Association between water consumption and body weight outcomes: A systematic review. Am. J. Clin. Nutr..

[19] Pan A., Malik V.S., Hao T., Willett W.C., Mozaffarian D., Hu F.B. (2013). Changes in water and beverage intake and long-term weight changes: Results from three prospective cohort studies. Int. J. Obes..

[20] Segui-Gomez M., de la Fuente C., Vazquez Z., de Irala J., Martinez-Gonzalez M.A. (2006). Cohort profile: The ‘Seguimiento Universidad de Navarra’ (SUN) study. Int. J. Epidemiol..

[21] Willett W. (2013). Nutritional Epidemiology.

[22] De la Fuente-Arrillaga C., Ruiz Z.V., Bes-Rastrollo M., Sampson L., Martinez-Gonzalez M.A. (2010). Reproducibility of an ffq validated in spain. Public Health Nutr..

[23] Fernandez-Ballart J.D., Pinol J.L., Zazpe I., Corella D., Carrasco P., Toledo E., Perez-Bauer M., Martinez-Gonzalez M.A., Salas-Salvado J., Martin-Moreno J.M. (2010). Relative validity of a semi-quantitative food-frequency questionnaire in an elderly Mediterranean population of Spain. Br. J. Nutr..

[24] Sayon-Orea C., Bes-Rastrollo M., Nunez-Cordoba J.M., Basterra-Gortari F.J., Beunza J.J., Martinez-Gonzalez M.A. (2011). Type of alcoholic beverage and incidence of overweight/obesity in a Mediterranean cohort: The SUN project. Nutrition.

[25] Bes-Rastrollo M. (2005). Validation of self-reported weight and body mass index of the participants of a cohort of university graduates. Rev. Esp. Obes..

[26] Ainsworth B.E., Haskell W.L., Herrmann S.D., Meckes N., Bassett D.R., Tudor-Locke C., Greer J.L., Vezina J., Whitt-Glover M.C., Leon A.S. (2011). 2011 compendium of physical activities: A second update of codes and MET values. Med. Sci. Sports Exerc..

[27] Martinez-Gonzalez M.A., Lopez-Fontana C., Varo J.J., Sanchez-Villegas A., Martinez J.A. (2005). Validation of the Spanish version of the physical activity questionnaire used in the Nurses’ Health Study and the Health Professionals’ follow-up Study. Public Health Nutr..

[28] Trichopoulou A., Costacou T., Bamia C., Trichopoulos D. (2003). Adherence to a Mediterranean diet and survival in a Greek population. N. Engl. J. Med..

[29] Hu F.B., Stampfer M.J., Manson J.E., Rimm E., Colditz G.A., Rosner B.A., Hennekens C.H., Willett W.C. (1997). Dietary fat intake and the risk of coronary heart disease in women. N. Engl. J. Med..

[30] Frankenfield D., Roth-Yousey L., Compher C. (2005). Comparison of predictive equations for resting metabolic rate in healthy nonobese and obese adults: A systematic review. J. Am. Diet. Assoc..

[31] Wannamethee S.G., Field A.E., Colditz G.A., Rimm E.B. (2004). Alcohol intake and 8-year weight gain in women: A prospective study. Obes. Res..

[32] Wannamethee S.G., Shaper A.G. (2003). Alcohol, body weight, and weight gain in middle-aged men. Am. J. Clin. Nutr..

[33] Alcacera M.A., Marques-Lopes I., Fajo-Pascual M., Puzo J., Blas Perez J., Bes-Rastrollo M., Martinez-Gonzalez M.A. (2008). Lifestyle factors associated with BMI in a Spanish graduate population: The SUN Study. Obes. Facts.

[34] Wang L., Lee I.M., Manson J.E., Buring J.E., Sesso H.D. (2010). Alcohol consumption, weight gain, and risk of becoming overweight in middle-aged and older women. Arch. Intern. Med..

[35] Vadstrup E.S., Petersen L., Sorensen T.I., Gronbaek M. (2003). Waist circumference in relation to history of amount and type of alcohol: Results from the Copenhagen City Heart Study. Int. J. Obes. Relat. Metab. Disord. J. Int. Assoc. Study Obes..

[36] Jimenez-Pavon D., Cervantes-Borunda M.S., Diaz L.E., Marcos A., Castillo M.J. (2015). Effects of a moderate intake of beer on markers of hydration after exercise in the heat: A crossover study. J. Int. Soc. Sports Nutr..

[37] Halkjaer J., Tjonneland A., Thomsen B.L., Overvad K., Sorensen T.I. (2006). Intake of macronutrients as predictors of 5-y changes in waist circumference. Am. J. Clin. Nutr..

[38] Sanchez-Villegas A., Toledo E., Bes-Rastrollo M., Martin-Moreno J.M., Tortosa A., Martinez-Gonzalez M.A. (2009). Association between dietary and beverage consumption patterns in the SUN (Seguimiento Universidad de Navarra) cohort study. Public Health Nutr..

[39] Monteiro R., Soares R., Guerreiro S., Pestana D., Calhau C., Azevedo I. (2009). Red wine increases adipose tissue aromatase expression and regulates body weight and adipocyte size. Nutrition.

[40] Fischer-Posovszky P., Kukulus V., Tews D., Unterkircher T., Debatin K.M., Fulda S., Wabitsch M. (2010). Resveratrol regulates human adipocyte number and function in a Sirt1-dependent manner. Am. J. Clin. Nutr..

[41] Cordain L., Bryan E.D., Melby C.L., Smith M.J. (1997). Influence of moderate daily wine consumption on body weight regulation and metabolism in healthy free-living males. J. Am. Coll. Nutr..

[42] Olsen N.J., Heitmann B.L. (2009). Intake of calorically sweetened beverages and obesity. Obes. Rev. Off. J. Int. Assoc. Study Obes..

[43] Daniels M.C., Popkin B.M. (2010). Impact of water intake on energy intake and weight status: A systematic review. Nutr. Rev..

[44] Stookey J.D., Constant F., Gardner C.D., Popkin B.M. (2007). Replacing sweetened caloric beverages with drinking water is associated with lower energy intake. Obesity.

[45] Stookey J.D., Constant F., Popkin B.M., Gardner C.D. (2008). Drinking water is associated with weight loss in overweight dieting women independent of diet and activity. Obesity.

[46] Boschmann M., Steiniger J., Franke G., Birkenfeld A.L., Luft F.C., Jordan J. (2007). Water drinking induces thermogenesis through osmosensitive mechanisms. J. Clin. Endocrinol. Metab..

[47] Pan A., Malik V.S., Schulze M.B., Manson J.E., Willett W.C., Hu F.B. (2012). Plain-water intake and risk of type 2 diabetes in young and middle-aged women. Am. J. Clin. Nutr..

[48] Ebbeling C.B., Feldman H.A., Osganian S.K., Chomitz V.R., Ellenbogen S.J., Ludwig D.S. (2006). Effects of decreasing sugar-sweetened beverage consumption on body weight in adolescents: A randomized, controlled pilot study. Pediatrics.

[49] Tate D.F., Turner-McGrievy G., Lyons E., Stevens J., Erickson K., Polzien K., Diamond M., Wang X., Popkin B. (2012). Replacing caloric beverages with water or diet beverages for weight loss in adults: Main results of the Choose Healthy Options Consciously Everyday (CHOICE) randomized clinical trial. Am. J. Clin. Nutr..

[50] Piernas C., Tate D.F., Wang X., Popkin B.M. (2013). Does diet-beverage intake affect dietary consumption patterns? Results from the Choose Healthy Options Consciously Everyday (CHOICE) randomized clinical trial. Am. J. Clin. Nutr..

[51] de Ruyter J.C., Olthof M.R., Seidell J.C., Katan M.B. (2012). A trial of sugar-free or sugar-sweetened beverages and body weight in children. N. Engl. J. Med..

[52] Peters J.C., Wyatt H.R., Foster G.D., Pan Z., Wojtanowski A.C., Vander Veur S.S., Herring S.J., Brill C., Hill J.O. (2014). The effects of water and non-nutritive sweetened beverages on weight loss during a 12-week weight loss treatment program. Obesity.

[53] Bes-Rastrollo M., Sanchez-Villegas A., Gomez-Gracia E., Martinez J.A., Pajares R.M., Martinez-Gonzalez M.A. (2006). Predictors of weight gain in a Mediterranean cohort: The Seguimiento Universidad de Navarra Study 1. Am. J. Clin. Nutr..

[54] Rajpathak S.N., Rimm E.B., Rosner B., Willett W.C., Hu F.B. (2006). Calcium and dairy intakes in relation to long-term weight gain in US men. Am. J. Clin. Nutr..

[55] Louie J.C., Flood V.M., Hector D.J., Rangan A.M., Gill T.P. (2011). Dairy consumption and overweight and obesity: A systematic review of prospective cohort studies. Obes. Rev. Off. J. Int. Assoc. Study Obes..

[56] Chen M., Pan A., Malik V.S., Hu F.B. (2012). Effects of dairy intake on body weight and fat: A meta-analysis of randomized controlled trials. Am. J. Clin. Nutr..

[57] Bernstein A.M., de Koning L., Flint A.J., Rexrode K.M., Willett W.C. (2012). Soda consumption and the risk of stroke in men and women. Am. J. Clin. Nutr..

[58] Guasch-Ferre M., Babio N., Martinez-Gonzalez M.A., Corella D., Ros E., Martin-Pelaez S., Estruch R., Aros F., Gomez-Gracia E., Fiol M. (2015). Dietary fat intake and risk of cardiovascular disease and all-cause mortality in a population at high risk of cardiovascular disease. Am. J. Clin. Nutr..

